# Optimisation of the fermentation media to enhance the production of the bioactive isomer of vitamin menaquinone-7

**DOI:** 10.1007/s00449-022-02752-6

**Published:** 2022-07-21

**Authors:** Neha Lal, Mostafa Seifan, Aydin Berenjian

**Affiliations:** 1 School of Engineering, The University of Waikato, Hamilton, 3240 New Zealand; 2 Department of Agricultural and Biological Engineering, Pennsylvania State University, 221 Agricultural Engineering Building, University Park, PA 16802 USA

**Keywords:** Menaquinone-7 isomers, Bioactivity, Fermentation, Media optimisation

## Abstract

Menaquinone-7 (MK-7) offers significant health benefits; however, only the all-*trans* form is biologically active. MK-7 produced through fermentation can occur as all-*trans* and *cis* isomers, and the therapeutic value of the resulting MK-7 is exclusively determined by the quantity of the all-*trans* isomer. Therefore, this study aimed to investigate the effect of the media composition on the isomer profile obtained from fermentation and determine the optimum media combination to increase the concentration of the all-*trans* isomer and diminish the production of *cis* MK-7. For this purpose, design of experiments (DOE) was used to screen the most effective nutrients, and a central composite face-centred design (CCF) was employed to optimise the media components. The optimum media consisted of 1% (*w/v*) glucose, 2% (*w/v*) yeast extract, 2% (*w/v*) soy peptone, 2% (*w/v*) tryptone, and 0.1% (*w/v*) CaCl_2_. This composition resulted in an average all-*trans* and *cis *isomer concentration of 36.366 mg/L and 1.225 mg/L, respectively. In addition, the optimised media enabled an all-*trans* isomer concentration 12.2-fold greater and a *cis* isomer concentration 2.9-fold less than the unoptimised media. This study was the first to consider the development of an optimised fermentation media to enhance the production of the bioactive isomer of MK-7 and minimise the concentration of the inactive isomer. Furthermore, this media is commercially promising, as it will improve the process productivity and reduce the costs associated with the industrial fermentation of the vitamin.

## Introduction

**Fig. 1:**
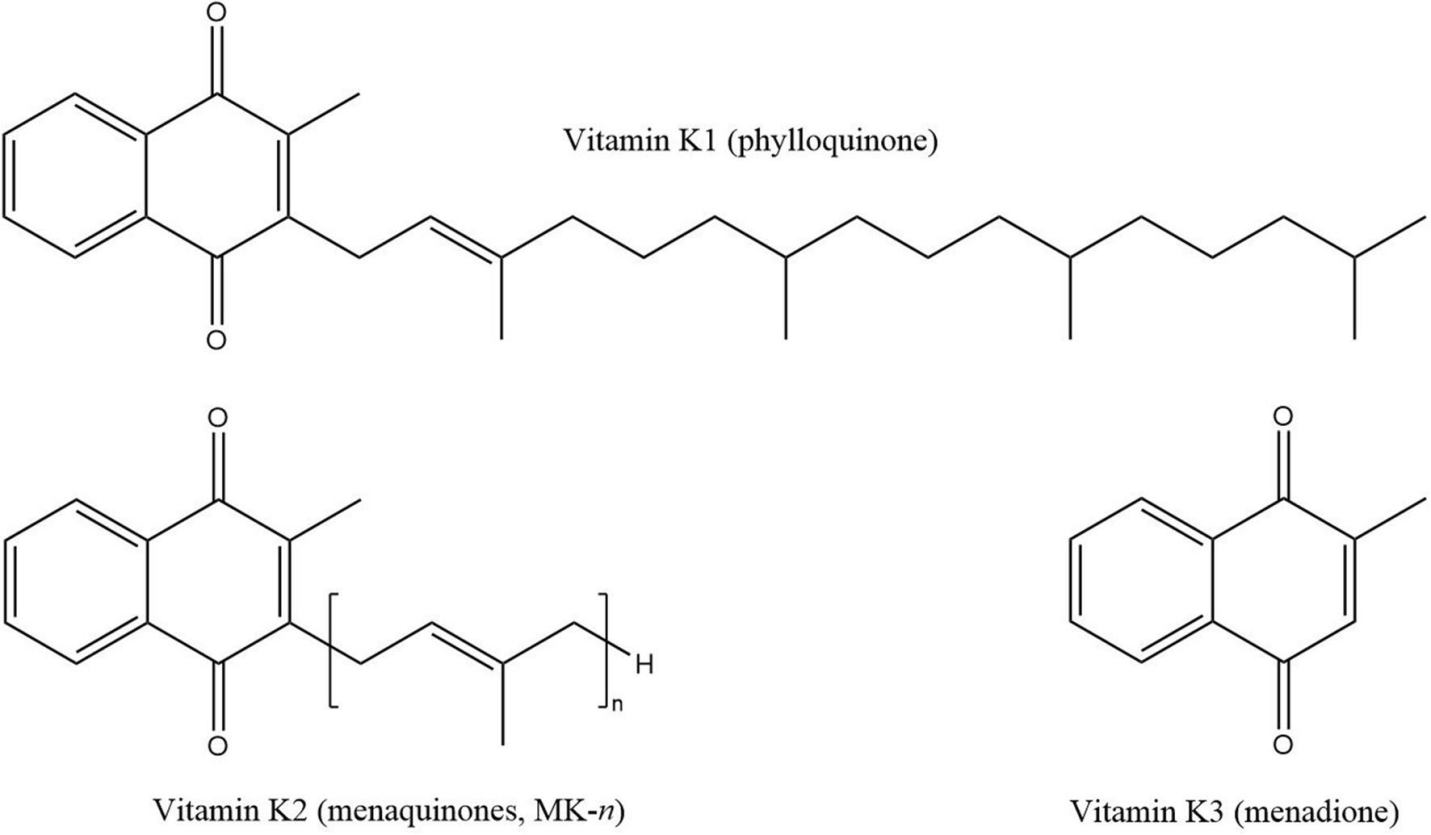
The different forms of vitamin K and their chemical structure (adapted from Szterk et al. [8])

Vitamin K is a fat-soluble vitamin first discovered in 1929 by the Danish nutritional biochemist Carl Peter Henrik Dam as an antihaemorrhagic factor capable of correcting dietary-induced bleeding disorders in chicks [1–3]. The vitamin K series consists of a group of compounds that contain a 2-methyl-1,4-naphthoquinone moiety (menadione) but differ in the structure of a lateral isoprenoid chain at the 3-position (Fig. 1) [4]. The length and degree of saturation of the isoprenoid side chain influence the properties of the various forms of vitamin K [5–7].

Vitamin K1 (phylloquinone) and vitamin K2 (menaquinones) are the two naturally occurring forms of vitamin K [9, 10]. Phylloquinone (PK) is a single compound and is the dominant source of vitamin K in the diet and can be obtained from green vegetables, vegetable oils, and products derived from these plant oils [5, 11, 12]. Whereas menaquinones (MK) are primarily of microbial origin and comprise side chains of varying length and degree of saturation; this is described by the general representation MK-*n*, where *n* denotes the number of unsaturated isoprenoid units in a chain, which is typically between four and thirteen [5, 8, 13]. The intestinal microbiota also contribute to the synthesis of MK in the human body, and MK-4, the most common isoform in humans, can also be produced from the tissue-specific conversion of PK [14].

All forms of vitamin K are involved in the blood coagulation pathway and haemostasis; however, numerous studies have established that the potential health gains of vitamin K extend well beyond the activation of hepatic coagulation factors. In particular, vitamin K intake has been associated with improved bone and cardiovascular health [5, 9, 15–22]. In addition, several more recent investigations have established other possible functions and health benefits of vitamin K, specifically, vitamin K2, which include the prevention of cancer, the suppression of Parkinson’s disease, assisting the functional recovery of the liver, and decreasing the risk of type 2 diabetes mellitus, chronic kidney disease, immune disorders, neurological disease, and obesity [21, 23–30]. Furthermore, it has been suggested that reduced vitamin K status is a possible modifiable risk factor for severe coronavirus disease 2019 (COVID-19) and may be linked to manifestations of COVID-19 and comorbidities related to the acute form of the disease [31, 32]. Hence, vitamin K supplementation will likely reduce the morbidity and mortality associated with COVID-19.

Of the various forms of vitamin K, MK-7 is the most notable and provides the greatest health benefits owing to its longer half-life in the body and superior extrahepatic availability [22, 33]. MK-7 supplementation can also facilitate anticoagulant therapy involving vitamin K antagonists, as low doses of MK-7 supplements can help improve anticoagulant management in patients [14]. Although, due to its low concentration in limited food products, obtaining adequate levels of MK-7 from regular food items is not feasible [34, 35]. Therefore, in light of the various health advantages of MK-7, the development of nutritional supplements and functional food products to complement natural food sources and improve the dietary intake of MK-7 has become increasingly widespread.

**Fig. 2:**
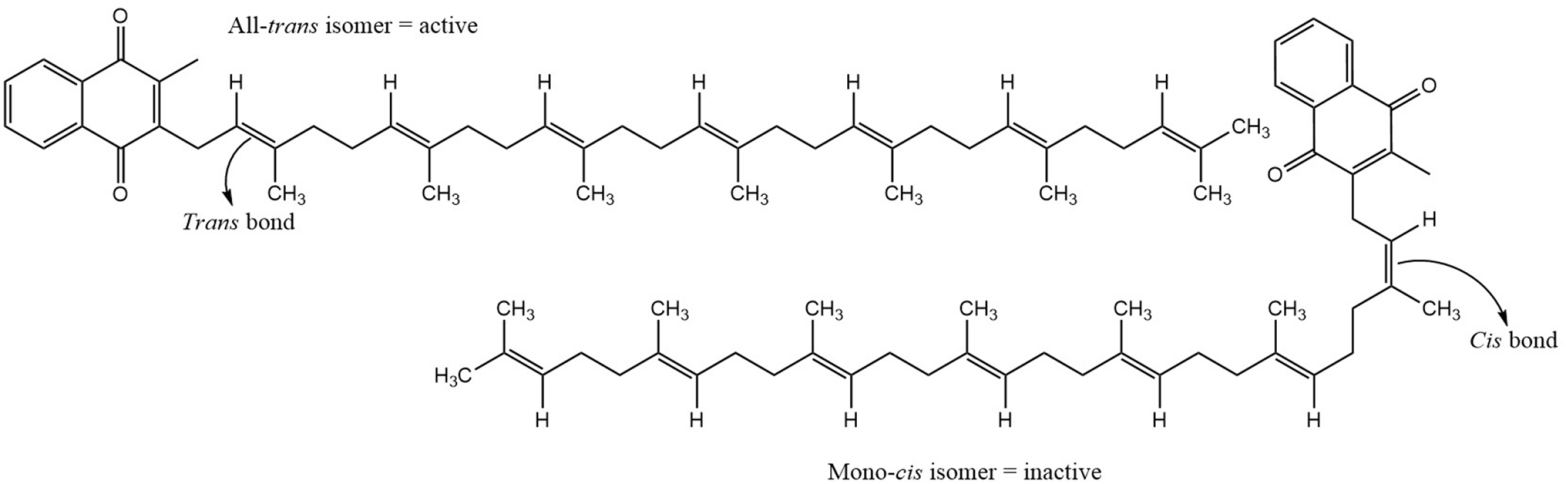
The chemical structure of *cis* and *trans* MK-7 isomers (adapted from Lal and Berenjian [40])

However, it is essential to note that, like most biological molecules, MK-7 can exist as geometric isomers, of which only the naturally occurring all-*trans* isomer is bioactive [6, 8, 36]. The double bond arrangement in the isoprenoid side chain of MK-7 molecules determines its shape and, consequently, its biological activity [6, 37]. The all-*trans* form of MK-7 has a linear molecular structure (Fig. 2), as all the double bonds in its side chain have the *trans* configuration [38]. Whereas in the *cis* isomers, the presence of one or more double bonds in the *cis* arrangement creates a bend in the isoprenoid chain, and this causes the *cis* isomers to adopt a non-linear structure (Fig. 2) [38]. The altered molecular structure of the *cis* isomers compromises their ability to carry out their biological function, and it has been demonstrated that the *cis* forms of vitamin K merely exhibit 1% of the biological activity of the all-*trans* isomer [6, 37, 39]. The bioactivity of MK-7 isomers is an important consideration from a health, nutritional, and therapeutic perspective.

The concept of MK-7 isomers is a relatively recent and emerging area of interest; thus, insufficient research has been conducted on this aspect, and a great deal is yet to be explored. MK-7 can be produced from fermentation [15, 41] or through chemical reaction methods [13, 42–45], and the isomer composition that is obtained is dependent on a variety of factors, primarily the methods used for the synthesis of the vitamin and purification of the post-reaction mixture [6, 8, 36, 38]. The molecular structure of MK-7 comprises seven double bonds, and in the all-*trans* isomer, all seven double bonds have the *trans* configuration. Individual double bonds in the isoprenoid chain of the molecule can also adopt the *cis* arrangement, and various *cis/trans* isomers, with different combinations of double bonds in the *cis* and *trans* configurations, may be attainable. However, the stability of the different isomers is likely to vary depending on the organisation of double bonds in the isoprenoid chain, which results in diverse molecular structures and shapes, as some forms of *cis* MK-7 may be less energetically favourable and are, therefore, less likely to exist than other more stable conformations.

Moreover, the number and type of different *cis* isomers that can potentially be achieved are ambiguous, as very few studies have considered this aspect and often present conflicting information. The majority of investigations that have focused on analysing the MK-7 isomer composition have explored dietary supplements or similar formulations [6, 8, 36, 46], and thus far, studies examining fermented samples have not been carried out. Additionally, the number and type of *cis* isomers that can be obtained from fermentation are unknown, as although fermentation is believed to produce the all-*trans* isomer selectively, the MK-7 isomer profile of fermented samples is yet to be elucidated.

The fermentation-based synthesis of MK-7 is superior, as most consumers prefer naturally derived products, which hence have a greater demand than synthetic formulations. Fermentation can also naturally enhance the nutrient profile and sensory characteristics of various products, thereby increasing their appeal to consumers [47, 48]. Furthermore, microbial fermentation is a more sustainable process for the large-scale production of MK-7, and the use of natural production methods can help satisfy the market demand and sustainable development goals [49]. Many Gram-positive and Gram-negative bacterial strains can synthesise MK, which function as electron carriers in the respiratory chain [5, 23, 50]. Both wild-type and engineered microorganisms have been used for MK-7 production via fermentation of which *Bacillus* strains, lactic acid bacteria, and various other types of microorganisms, such as *Escherichia coli*, *Flavobacterium meningosepticum*, *Enterobacter agglomerans, Enterococcus faecium,* and *Serratia marcescens*, tend to be the most common [49]. Nevertheless, members of the *Bacillus* species, such as *Bacillus subtilis natto* [51], *Bacillus licheniformis* [52], and *Bacillus amylolyquifaciens* [53], are the most notable [54]. Of the several suitable strains, *B. subtilis natto* is considered to be the most ideal for the industrial production of MK-7 and is preferentially used for the manufacture of MK-7 supplements and functional food products, as it is generally recognised as safe (GRAS) and enables a high MK-7 yield [15, 55–60]. As a result, there are no safety concerns associated with the *B. subtilis natto* strain, and it is suitable for the production of microbial-derived MK-7 products that are intended for human consumption.

Numerous studies have explored and enhanced different aspects of the fermentation process in various contexts, including liquid-state fermentation (LSF) [55, 58, 60–63], solid-state fermentation (SSF) [64–69], and biofilm reactors [54, 70–76], to improve MK-7 production using *B. subtilis natto*. The fermentation media, in particular, has been of considerable interest, as the selection of carbon, nitrogen, and salt sources and presence of growth factors, vitamins, minerals, bioactives, and other essential nutrients play a crucial role in microbial growth and metabolism [77, 78], which ultimately influence the productivity of the fermentation process and the MK-7 yield. Recently, several investigations have focused on improving the MK-7 yield by optimising the fermentation media using DOE and response surface methodology (RSM) [54, 61, 62, 69, 79, 80]. This approach allows both the individual and interactive effects of nutrients to be considered to determine the optimum media composition for a specific fermentation process, unlike the conventional method in which components are varied and analysed independently. However, although fermentation medium engineering to improve MK-7 productivity has been the subject of many studies, the focus has predominantly been to enhance MK-7 production without regard to the proportion of the bioactive all-*trans* isomer obtained from fermentation under the investigated conditions. The isomer composition achieved through fermentation is a key aspect worthy of attention, as the effectiveness of MK-7 nutritional supplements and functional food products is only determined by the content of the all-*trans* isomer, and all other isomeric forms of the vitamin are essentially impurities that lack biological significance.

Therefore, the primary objective of this study was to investigate the effect of the media composition on the MK-7 isomer profile and determine the optimum combination of nutrients and their required concentrations to enhance the fermentation yield of the all-*trans* isomer and reduce the production of *cis* MK-7. As part of this process, ten different nutrient components, including carbon, nitrogen, and salt sources, were initially screened using a Plackett–Burman design (PBD) to identify the important nutritional factors that significantly impact the isomer concentration. A CCF design and RSM were then employed to optimise the concentration of the significant nutrients determined from the screening stage to develop the ideal fermentation media to enhance the production of the bioactive all-*trans* isomer and minimise the yield of the biologically insignificant *cis* isomer. This study will create new opportunities to develop an industrial fermentation process that targets the synthesis of the all-*trans* MK-7 isomer, which is commercially attractive, as it will refine the production process and decrease the related costs. This will be a valuable step forward in increasing the accessibility of biologically active fermented MK-7 supplements and functional food products to consumers, which will likely ameliorate the dietary intake of MK-7 and improve health outcomes.

## Materials and methods

### Chemicals and materials

The all-*trans* MK-7 analytical standard (98.1% purity) was purchased from ChromaDex (Los Angeles, CA, USA). Glucose was obtained from Ajax Finechem Pty Ltd (Taren Point, NSW, Australia), and yeast extract, peptone, tryptone, and soytone were acquired from Becton, Dickinson and Company (Franklin Lakes, NJ, USA). Glycerol, soy peptone, K_2_HPO_4_, methanol, 2-propanol, and *n*-hexane were purchased from Merck Millipore (Burlington, MA, USA). NaCl was obtained from a domestic supplier, and CaCl_2_ was acquired from Sigma-Aldrich (St. Louis, MO, USA). Nutrient agar plates were purchased from Fort Richard Laboratories (Auckland, New Zealand).

### Microorganism and inoculum preparation

The *B. subtilis natto* strain was prepared as described previously [61]. Briefly, the cells were cultivated in a liquid culture medium containing tryptone, yeast extract, and NaCl before streaking on nutrient agar plates. The plates were incubated at 37 °C for 48 h. The cells were then scraped off the plates and suspended in a sterilised saline solution (0.9% (*w/v*) NaCl). The suspension was subsequently placed in a water bath at 80 °C for 30 min to inactivate the vegetative cells and induce the production of spores before centrifuging (laboratory centrifuge, Sigma Laborzentrifugen GmbH, Osterode am Harz, Germany) at 3000 rpm for 10 min to remove the cell debris. The resulting spore suspension (4.8 × 10^6^ CFU/mL) was used as the inoculum for the fermentation experiments.

### Experimental design and statistical analysis

Ten different nutrients, which have been shown to enhance the MK-7 yield, were selected based on previous studies [54, 60–63, 69, 80, 81], and the effect of various carbon, nitrogen, and salt sources on isomer production was considered. Glucose and glycerol were the two carbon sources explored; yeast extract, soy peptone, peptone, tryptone, and soytone were selected as potent complex nitrogen sources; and K_2_HPO_4_, CaCl_2_, and NaCl were determined to be effective salt sources. All carbon and nitrogen sources were investigated in the range of 0–2% (*w/v*), while the salt sources were considered between 0–1% (*w/v*). These concentration spans were derived from the literature and preliminary experiments.

A PBD was implemented to examine the individual effects of the selected nutritional factors on the all-*trans* and *cis* MK-7 isomer concentrations achieved during fermentation. Each factor was considered at three levels (high, intermediate, and low). A CCF design was employed to optimise the significant variables identified from the screening step, and RSM was used to analyse the results. The experimental values were scaled factors, and the response was described by a quadratic equation. The MODDE 13 software (Sartorius, Gottingen, Germany) was used to create the design matrices, develop a model, and determine the optimum level of the media components to achieve the highest concentration of all-*trans* MK-7 and minimise the concentration of the *cis* isomer. The experimental data was then used to generate a second-order polynomial regression model (Eq. 1) for each response.

1 $$Y= {b}_{0}+\sum {b}_{i}{X}_{i}+\sum {b}_{ii}{X}_{i}^{2}+\sum {b}_{ij}{X}_{i}{X}_{j}$$

Where *Y* represents the all-*trans* or *cis* MK-7 isomer concentration; *b*_0_ is a constant term; *b*_*i*_, *b*_*ii*_, and b_*ij*_ are the coefficients of the linear, quadratic, and synergistic effects, respectively; and *X*_*i*_ and *X*_*j*_ correspond to the significant factors. The *R*^2^ value was used to express the quality of the fit for the developed regression models, and statistical significance was determined using the analysis of variance (ANOVA) test and accepted at *P* < 0.1.

### Fermentation procedure

For both the screening and optimisation experiments, the fermentation media was prepared according to the DOE plan and sterilised using an autoclave (TOMY SX-700E, Tokyo, Japan) at 121 °C for 20 min. Each sample was then inoculated with 5% (*v*/*v*) of the pre-prepared *B. subtilis natto* spore suspension. Fermentation was conducted aerobically at 37 °C under dynamic conditions (120 rpm) for six days. The inoculum volume and operating conditions were selected based on preliminary studies. A small fermentation volume (6 mL) was used to enable the extraction of the whole sample, as it allowed all of the MK-7 produced during fermentation to be analysed and eliminated any errors associated with sampling from the fermentation media.

### MK-7 extraction

MK-7 was extracted from the samples prior to analysis using a mixture of 2-propanol and *n*-hexane in the ratio of 1:2 (*v*/*v*) and a liquid-to-organic ratio of 1:4 (*v*/*v*) [61]. The mixture was vigorously shaken for 2 min using a vortex mixer and centrifuged (laboratory centrifuge, Sigma Laborzentrifugen GmbH, Osterode am Harz, Germany) at 3000 rpm for 10 min to separate the two phases. Afterwards, the upper hexane layer was separated from the aqueous phase and evaporated under a vacuum to recover the extracted MK-7.

### MK-7 analysis

MK-7 analysis was carried out using the method outlined by Berenjian et al. [35] with minor alterations to accommodate the requirements of the chromatography column used in this study. A Dionex high-performance liquid chromatography (HPLC) system (Thermo Fisher Scientific, Waltham, MA, USA) equipped with four pumps (P680), an automated sample injector (ASI-100), a thermostatted column compartment (TCC-100), and a photodiode array UV detector (UVD340U) was employed to determine the MK-7 isomer concentration in the fermented samples. A COSMOSIL Cholester packed column (100 mm × 2 mm × 2.5 µm; Nacalai Tesque Inc., Kyoto, Japan) operated at 40 °C was used to separate the compounds. Methanol, at a flow rate of 0.2 mL/min, was used as the mobile phase (isocratic elution), and the analytical wavelength, injection volume, autosampler temperature, and run-time were 248 nm, 10 µL, 10 °C, and 30 min, respectively. The Chromeleon 7 software (Thermo Fisher Scientific, Waltham, MA, USA) was used for data acquisition. The MK-7 calibration curve was linear between 0.1 mg/L and 50 mg/L (*R*^2^ = 0.99).

Liquid chromatography-mass spectrometry (LC–MS) techniques were applied to confirm the presence and verify the chromatographic retention times of the all-*trans* and *cis* MK-7 isomers. The method developed by Szterk et al. [8] was employed as a guide for the LC–MS analysis, and although the fundamental concept was similar, specific aspects of the procedure were tailored to suit the requirements of the LC–MS system that was used in the present study. The LC–MS platform consisted of a Dionex Ultimate 3000 ultra-high-performance liquid chromatography (UHPLC) system and a QExactive mass spectrometer with a HESI II source (Thermo Fisher Scientific, Waltham, MA, USA). The Thermo XCalibur 4.3 software (Thermo Fisher Scientific, Waltham, MA, USA) was used to control the system, and data handling was carried out using the Chromeleon 7.3 software (Thermo Fisher Scientific, Waltham, MA, USA). Separation by liquid chromatography was performed using the chromatographic conditions outlined above, except the injection volume was altered to 5 µL, and the run-time was extended to 37 min to adapt to the requirements of the LC–MS system. Data collection was carried out in the positive ionisation mode with an MS1 scan range of 150–1000 *m/z*, a resolution of 70,000, an AGC target of 3 × 10^6^, and a maximum injection time of 200 ms. The mass spectrometry (MS) data were analysed using the Thermo FreeStyle 1.6 software (Thermo Fisher Scientific, Waltham, MA, USA).

### Cell density and pH measurements

Bacterial growth was inferred from the cell density, which was determined by measuring the optical density (OD) at 600 nm using a UV–vis spectrophotometer (Shimadzu UV-1900, Kyoto, Japan) after appropriate dilution with distilled water. The pH was directly measured in the cultivation medium with a standard laboratory pH meter (CyberScan pH 100, Eutech Instruments, Paisley, UK).

## Results and discussion

### LC–MS analysis

Relative to traditional MK-7 analytical techniques, which do not consider the isomer composition of samples, the analysis and measurement of MK-7 isomers present various difficulties. The fundamental issue is the absence of specific analytical standards for the potential *cis* forms of the vitamin, as only the all-*trans* MK-7 standard is commercially available [8, 36]. As a result, the identification and quantification of *cis* MK-7 pose a challenge due to the lack of appropriate reference standards. In addition, very few studies have considered the concept of MK-7 isomers [6, 8, 36, 46, 82], and the primary focus of these investigations has been to analyse the isomer composition of MK-7 dietary supplements and other similar preparations. Therefore, the nature of the MK-7 isomer profile obtained from fermentation is unknown, as fermented samples are yet to be explored.

This study used the all-*trans* MK-7 reference standard to identify and quantify the all-*trans* and *cis* MK-7 isomers present in fermented samples. The retention time of the peaks in the analytical standard was used to identify the corresponding peaks in the samples, which consistently appeared at approximately 12.6 min, 19.7 min, and 22.1 min. The compounds pertaining to these peaks were speculated to be MK-7 isomers. Due to the unavailability of suitable reference standards, MS techniques were used to verify the identity of the isomers by confirming the presence of a peak at approximately 649.5 *m/z* (molecular mass of MK-7) in the MS data and comparing the mass spectra of the samples to that of the all-*trans* MK-7 standard. This approach was valid, as the compounds of interest are isomers and have the same molecular mass (approximately 649.5 *m/z*), and this, together with the mass spectra, can be used to ascertain whether the three chromatographic peaks represent MK-7 isomers. A similar strategy has been successfully demonstrated in the series of studies conducted by Szterk et al. [8], Szterk et al. [36], and Sitkowski et al. [6] for the analysis of MK-7 isomers present in dietary supplements.

**Fig. 3:**
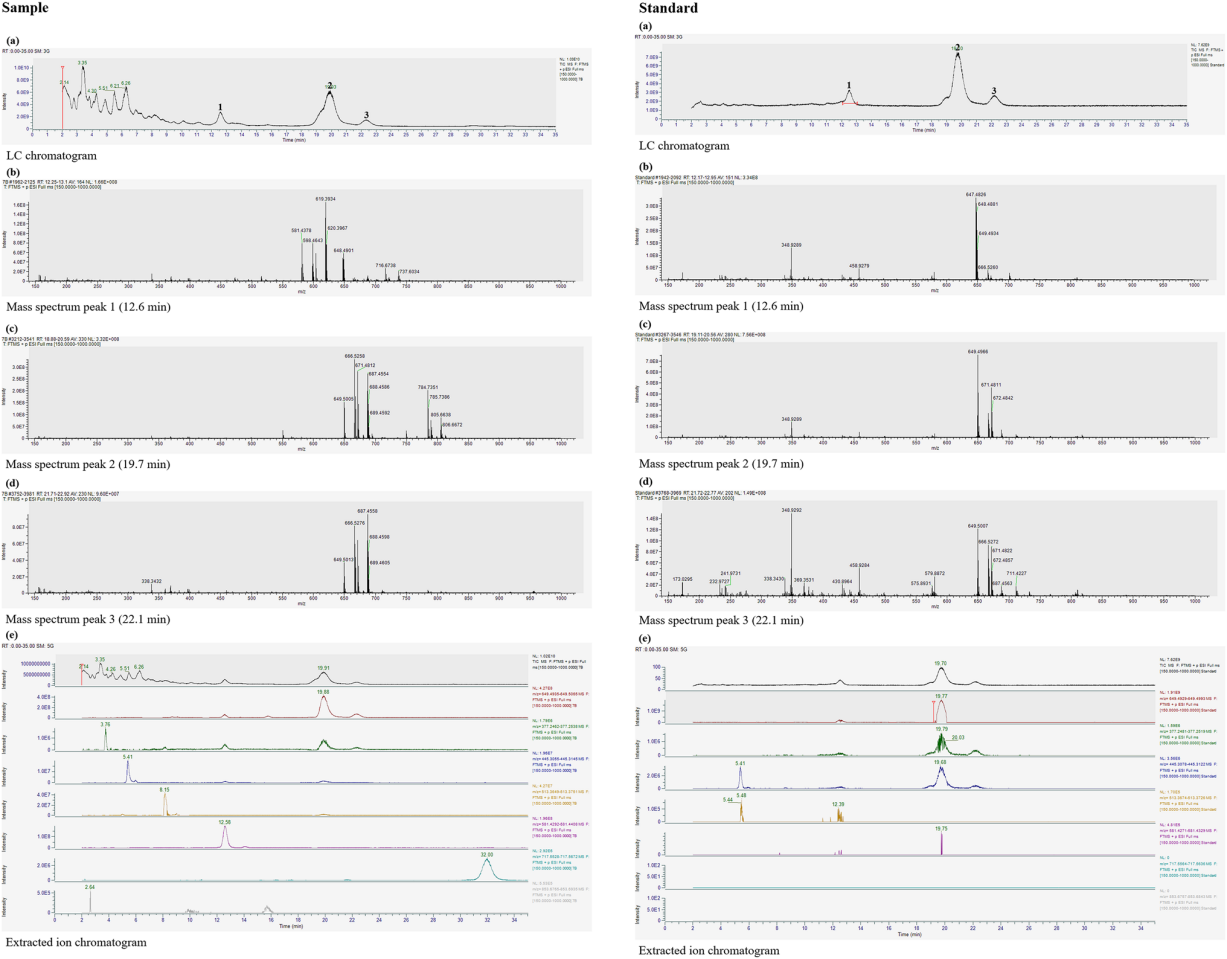
Liquid chromatography (LC) chromatograms **a**, MS data **b-d**, and extracted ion chromatograms **e** for an experimental sample and the all-*trans* MK-7 reference standard

The MS analysis determined that the compounds relating to the peaks at approximately 19.7 min and 22.1 min were MK-7 isomers, while the peak at approximately 12.6 min was not (Fig. 3). The MS data for the peaks at approximately 19.7 min and 22.1 min were comparable and showed a similar set of mass peaks at each retention time across all samples. The MS data for both peaks also showed a mass peak at approximately 649.5 *m/z*. Although other mass peaks, which may indicate impurities or other possible MK of different chain lengths, were observed at these time points, they were not as significant, and evaluation of the MS data obtained from the samples and standard was sufficient to establish that the compounds corresponding to the peaks at approximately 19.7 min and 22.1 min were MK-7 isomers. The chromatographic peak appearing at approximately 19.7 min was notably larger than that at approximately 22.1 min. Consequently, it is anticipated to correspond to the all-*trans* isomer, as all-*trans* MK-7 is the naturally occurring form of the vitamin and is, thus, likely to be produced in a greater quantity relative to the *cis* isomer, which is represented by the peak at approximately 22.1 min. A larger peak at approximately 19.7 min was also observed for the standard, which is a commercial sample of high purity (98.1% all-*trans* MK-7) and, as a result, contains a significantly greater proportion of the all-*trans* isomer.

From the LC–MS analysis of the fermented samples, it was also established that only a single *cis* MK-7 isomer is produced during fermentation (under the conditions employed). In contrast, it may be possible for samples of chemical or other origins to contain more than one *cis* MK-7 isomer, which may arise due to the synthesis procedures or purification techniques implemented in the production of the vitamin or vitamin product. Additionally, while these compounds are isomers, they have slightly different retention times owing to minor differences in their structure, resulting in their differing ability to move through the stereoselective column. The retention time of the all-*trans* and *cis* MK-7 isomers is consistent with the analytical standard and across a range of fermented samples, including samples from preliminary experiments that had different media components and compositions. It has also been established through these experiments that when employing the previously mentioned chromatography conditions, the relative retention time (RRT) of the *cis* isomer, which is the ratio of the retention time of the *cis* isomer to that of the all-*trans* isomer, is always constant (1.12) for all samples and is identical to that for the reference standard. Use of the RRT to distinguish the *cis* MK-7 isomer has been recommended in the USP Monograph [83] (RRT of 1.1) and demonstrated in the investigation carried out by Jedynak et al. [82] (RRT of 1.15). Although the suggested values for the RRT vary slightly between these sources and the present study, these minor differences are likely to be due to the different instruments, columns, solvents, chromatographic conditions, and analytical procedures that have been employed in each case to achieve separation. Therefore, the RRT is a reliable method to identify the all-*trans* and *cis* MK-7 isomers in fermented samples using HPLC, which is far more convenient and avoids the need to recurrently use LC–MS techniques for the analysis of samples in subsequent experiments.

### Screening study

The selected media components, consisting of different carbon, nitrogen, and salt sources, were initially screened using a PBD to determine the important nutritional factors that significantly affect the fermentation yield of the all-*trans* and *cis* isomers. The carbon and nitrogen sources were considered between 0–2% (*w/v*), while the salt sources were explored in the range of 0–1% (*w/v*). The concentration span of each nutrient was selected based on the literature and from the unfavourable results that were obtained from preliminary experiments, which examined higher nutrient concentrations (up to 10% (*w/v*) for the carbon and nitrogen sources and up to 2% (*w/v*) for the salt sources). The unsatisfactory results may be attributed to the high nutrient concentrations, which are likely to decrease the water activity and induce osmotic stress, consequently inhibiting cell growth, metabolism, and MK-7 production [53, 61]. Hence, it was decided to investigate lower nutrient concentrations in the screening study. Due to the large number of factors being investigated, it was not feasible to consider both the individual and interactive effects of the different nutrients. Therefore, only the individual effects of the media components on the isomer concentration were evaluated to ensure that the number of experimental runs was practical. The experimental design and the isomer concentrations are outlined in Table 1, and the statistical analysis is presented in Table 2.

**Table 1:** Experimental design and measured responses from the screening study

Nutrient factors (% *w/v*)											Measured responses (mg/L)	
Run	Glucose	Glycerol	Yeast extract	Soy peptone	Peptone	Tryptone	Soytone	K_2_HPO_4_	CaCl_2_	NaCl	All-*trans* MK-7 concentration	*Cis* MK-7 concentration
1	2	0	0	0	2	0	0	1	1	0	0.000	0.000
2	2	2	0	0	0	2	0	0	1	1	11.940	1.064
3	2	2	2	0	0	0	2	0	0	1	37.494	2.302
4	2	2	2	2	0	0	0	1	0	0	57.336	3.343
5	0	2	2	2	2	0	0	0	1	0	10.201	0.869
6	2	0	2	2	2	2	0	0	0	1	40.551	2.480
7	0	2	0	2	2	2	2	0	0	0	53.771	3.512
8	2	0	2	0	2	2	2	1	0	0	43.654	2.723
9	2	2	0	2	0	2	2	1	1	0	21.600	1.285
10	0	2	2	0	2	0	2	1	1	1	0.666	0.000
11	0	0	2	2	0	2	0	1	1	1	19.768	1.330
12	2	0	0	2	2	0	2	0	1	1	21.402	1.385
13	0	2	0	0	2	2	0	1	0	1	5.246	0.609
14	0	0	2	0	0	2	2	0	1	0	20.425	1.481
15	0	0	0	2	0	0	2	1	0	1	12.763	0.996
16	0	0	0	0	0	0	0	0	0	0	0.511	0.000
17	1	1	1	1	1	1	1	0.5	0.5	0.5	18.32	1.486
18	1	1	1	1	1	1	1	0.5	0.5	0.5	19.118	1.584
19	1	1	1	1	1	1	1	0.5	0.5	0.5	30.664	2.387

**Table 2:** Statistical analysis of the variables from the screening study

All-*trans* MK-7 concentration			
Term	Coefficient	Standard error	*P*-value
Constant	22.391	2.276	9.592e-06
Glucose	6.914	2.480	0.024
Glycerol	2.449	2.480	0.352
Yeast extract	6.429	2.480	0.032
Soy peptone	7.341	2.480	0.018
Peptone	− 0.397	2.480	0.877
Tryptone	4.786	2.480	0.090
Soytone	4.139	2.480	0.134
K_2_HPO_4_	− 2.204	2.480	0.400
CaCl_2_	− 9.083	2.480	0.006
NaCl	− 3.604	2.480	0.184

*Cis* MK-7 concentration			
Term	Coefficient	Standard error	*P*-value
Constant	1.5177	0.1541	9.5025e-06
Glucose	0.3616	0.1679	0.0634
Glycerol	0.1618	0.1679	0.3634
Yeast extract	0.3548	0.1679	0.0675
Soy peptone	0.4388	0.1679	0.0310
Peptone	− 0.0139	0.1679	0.9359
Tryptone	0.3493	0.1679	0.0711
Soytone	0.2493	0.1679	0.1759
K_2_HPO_4_	− 0.1754	0.1679	0.3266
CaCl_2_	− 0.5344	0.1679	0.0129
NaCl	− 0.1904	0.1679	0.2896

*R*^2^ = 0.855; *R*^2^ (adj.) = 0.673; significance accepted at *P* < 0.1

*R*^2^ = 0.818; *R*^2^ (adj.) = 0.590; significance accepted at *P* < 0.1

### Carbon sources

Carbon sources are essential for MK-7 synthesis, as the pathway for producing the isoprene side chain and quinone skeleton (1,4-naphthoquinone) of MK-7 relies on the presence of carbon sources in the fermentation media [69]. Numerous carbon sources, including glucose, glycerol, sucrose, molasses, inulin, mannose, starch, dextrose, fructose, corn syrup, and maltose, have been extensively examined in previous investigations focusing on improving the MK-7 yield in a range of contexts and applications, such as SSF, LSF, biofilm reactors, and the development of MK-7 enriched nutraceuticals, functional foods, and animal feed [15, 53, 54, 58, 61–63, 69, 70, 72, 73, 81, 84–86]. However, of these, the majority of studies have determined glucose and glycerol to be the most effective at enhancing MK-7 production. Therefore, simply glucose and glycerol were considered in the screening process.

Of the two evaluated carbon sources, only glucose had a significant effect on the isomer concentration (*P* < 0.1). In addition, the positive coefficient for glucose for the all-*trans* and *cis* MK-7 isomer concentrations implies that glucose has a significant positive effect on the two responses and, thus, enhances the concentration of both isomers. Considering that the all-*trans* isomer is the biologically active form of the vitamin, it is beneficial to increase the production of the all-*trans* isomer, whereas, in comparison, *cis* MK-7 has very little or no bioactivity; hence, it is necessary to minimise its fermentation yield.

It has been suggested that glucose promotes cell growth, while glycerol, although not beneficial for cell growth, facilitates MK-7 biosynthesis and secretion in *B. subtilis natto* and increases MK-7 productivity on a per-cell basis [23, 51, 62, 71, 72, 87]. Therefore, glycerol has commonly been used to enhance the MK-7 yield and is often reported as the most efficient carbon source in MK-7 fermentation [53, 61, 62, 69, 81, 88]. However, various investigations, including more recent studies involving biofilm reactors, have demonstrated glucose to be an effective carbon source for MK-7 biosynthesis [54, 71, 73]. Interestingly, only glucose has a significant positive effect on the isomer concentration in the present study. A possible explanation for this could be that since glucose, compared to glycerol, is a more preferable and readily metabolisable carbon source for *B. subtilis* strains, it facilitates rapid microbial growth and, consequently, has a more substantial contribution to the MK-7 yield, as MK-7 production is partially growth-associated [35, 54, 58, 61, 63, 74, 84, 87–89]. Moreover, prior investigations have only explored the overall fermentation yield of MK-7 without any regard to the isomer profile; thus, the impact of glucose and glycerol may differ when considering MK-7 isomers.

In light of the aims of this study, glucose acts to enhance the production of all-*trans* MK-7, which is favourable; however, it also increases the concentration of the *cis* isomer, which is undesirable. Therefore, it is necessary to balance the glucose concentration in the fermentation media to regulate isomer production.

### Nitrogen sources

The selection of nitrogen sources in the fermentation media plays a crucial role in microbial growth and metabolism and the production of proteins involved in the cellular respiration processes of *B. subtilis*, such as haem [54, 61, 88]. The nature of the nitrogen source tends to influence the fermentation yield of MK-7, as inorganic and complex nitrogen sources supply different concentrations and types of amino acids, which may act as precursors or feedback inhibitors of the shikimate pathway that is involved in the synthesis of MK-7 and other MK [61].

Several inorganic and complex nitrogen sources, including NaNO_3_, (NH_4_)_2_SO_4_, KNO_3_, urea, soy protein, yeast extract, malt extract, peptone, tryptone, soy peptone, and soytone, have been considered in earlier studies focusing on MK-7 fermentation [15, 23, 51, 54, 58, 60, 62, 63, 69, 81, 87, 88]. Essentially, it has been determined that complex nitrogen sources are superior to inorganic nitrogen sources and are more efficient in supporting *B. subtilis* growth, various microbial processes, and MK-7 synthesis, as they tend to provide an assortment of amino acids and other essential growth factors, such as polypeptides and coenzymes [60, 90, 91]. In particular, yeast extract, soy peptone, peptone, tryptone, and soytone have frequently been employed in MK-7 fermentation studies and are deemed to be the most efficacious complex nitrogen sources for enhancing the yield of the vitamin [15, 54, 58, 62, 63, 85, 88]. Accordingly, only complex nitrogen sources, consisting of yeast extract, soy peptone, peptone, tryptone, and soytone, were included in the screening study.

From the five nitrogen sources that were screened, all but peptone and soytone had a significant effect on the yield of both isomers (*P* < 0.1). Yeast extract, soy peptone, and tryptone all have positive coefficients for the two isomers, suggesting that all of these complex nitrogen sources have a significant positive effect on the concentration of the all-*trans* and *cis* isomers. Since only the all-*trans* isomer sustains biological activity, it is advantageous to improve its productivity and reduce the proportion of the *cis* isomer achieved during fermentation.

The findings of the present study are supported by observations from prior investigations, which have commonly identified yeast extract, soy peptone, and tryptone as potent nitrogen sources that enhance MK-7 production [54, 58, 61–63, 80, 88, 92]. Yeast extract, soy peptone, and tryptone provide a broad spectrum of nitrogen compounds, such as short-chained and simple amino acids, that are easily metabolised by *B. subtilis* strains [54]. This is important from the perspective of MK-7 biosynthesis, which is an elaborate process that comprises several interrelated steps, namely the shikimate pathway to form the aromatic ring, the methylerythritol 4-phosphate (MEP) and isopentenyl diphosphate route to produce the isoprenoid tail, and the MK pathway in which the two separately synthesised structures are combined to generate MK-7 [23, 93–95]. The complexity of the MK-7 biosynthetic pathway is likely to have high energy requirements, mainly due to the need to produce the amino acids involved in MK-7 synthesis. The energy requirements can be reduced if the bacteria are supplied with the necessary amino acids, eliminating the need for their synthesis and thereby accelerating MK-7 production.

It has also been established that yeast extract and soy peptone support microbial growth and metabolism and have an interactive influence on MK-7 productivity [58, 61, 96], possibly due to the beneficial effect of the combined range and concentration of amino acids and nutrients, and are, thus, often used in conjunction in MK-7 fermentation [35, 58, 61, 88]. Moreover, the yeast extract, soy peptone, and tryptone used in this study have different origins (the yeast extract is derived from autolysed yeast cells, the soy peptone is papain-digested, and the tryptone is a pancreatic digest of casein) and, hence, are likely to supply a diverse range and concentration of amino acids, growth factors, and other vital compounds that synergistically promote microbial growth and metabolism and, ultimately, MK-7 biosynthesis.

Considering the bioactivity of the two MK-7 isomers and the objectives of this investigation, the effect of yeast extract, soy peptone, and tryptone on the production of all-*trans* MK-7 is beneficial, whereas their impact on the *cis* isomer concentration is not ideal. Consequently, it is important to ensure that the fermentation media contains the correct amounts of these nitrogen sources to control isomer production.

### Salt sources

Salts are an important source of trace and essential elements that perform vital functions during cell growth, metabolism, and product formation [91]. Common trace elements include iron (Fe^2+^ and Fe^3+^), zinc (Zn^2+^), potassium (K^+^), magnesium (Mg^2+^), manganese (Mn^2+^), molybdenum (Mo^2+^), sodium (Na^+^), cobalt (Co^2+^), calcium (Ca^2+^), and copper (Cu^2+^), which are often supplied in small quantities in the form of mineral salts [91].

Previous experiments have explored the effect of various salts, such as K_2_HPO_4_, CaCl_2_, MgSO_4_.7H_2_O, MnCl_2_.4H_2_O, and NaCl, on MK-7 production, and of these, K_2_HPO_4_, CaCl_2_, and NaCl are deemed to be the most effective [35, 51, 61, 63, 69, 80, 85, 86, 90, 92]. Therefore, K_2_HPO_4_, CaCl_2_, and NaCl were considered in the screening study, and only CaCl_2_ had a significant effect on MK-7 isomer production (*P* < 0.1). Additionally, its negative coefficient indicates that CaCl_2_ has a significant adverse effect on the isomer concentration. This is not advantageous for the all-*trans* MK-7 yield, as the goal of this study is to maximise its production; conversely, CaCl_2_ has a favourable influence on the *cis* isomer concentration, as the intention is to minimise its synthesis during fermentation.

The majority of previous investigations have reported that K_2_HPO_4_ is an important phosphate source that facilitates the production of primary and secondary metabolites and has a positive effect on MK-7 production [34, 35, 51, 61, 63, 80, 88], which is not consistent with the observations from the present study. However, prior investigations have exclusively focused on the overall fermentation yield of MK-7 and have not considered the proportion of MK-7 isomers that are obtained; therefore, K_2_HPO_4_ may have an insignificant effect on the production of MK-7 isomers. Inversely, it has been demonstrated in the study conducted by Singh et al. [69] that CaCl_2_ has a significant positive effect on MK-7 production, and it has also been established that an adequate concentration of Ca^2+^ benefits MK-7 biosynthesis [23]. It is important to note that in the present study, while it has been determined that CaCl_2_ has a significant effect on the MK-7 isomer yield, it acts to decrease the concentration of both isomers. The contrasting effect of CaCl_2_ in the two investigations is likely to be a result of the differing aims of each study, as while CaCl_2_ may have a positive effect on MK-7 production holistically, it may have the opposite effect when considering the MK-7 isomer profile.

Despite the negative impact of CaCl_2_ on the MK-7 isomer concentration, its presence in the fermentation media is imperative to decrease the yield of the *cis* isomer. Therefore, given the biological significance of the different MK-7 compounds and the purpose of this study, the concentration of CaCl_2_ needs to be optimised to reduce the synthesis of the *cis* isomer without significantly diminishing the productivity of the all-*trans* isomer.

### Optimisation of the fermentation media

The screening study concluded that glucose, yeast extract, soy peptone, tryptone, and CaCl_2_ significantly impact MK-7 isomer production. The presence of glucose, yeast extract, soy peptone, and tryptone is advantageous to improve the all-*trans* MK-7 isomer concentration; however, they also increase the concentration of *cis* MK-7, which is unfavourable. Whereas CaCl_2_ decreases the production of all-*trans* MK-7, which is not ideal but it is also beneficial, as it reduces the *cis* isomer yield. As a result, given the synonymous nature of the effect of the significant nutrients on the two responses, it is likely not to be possible to eliminate the synthesis of *cis* MK-7; nevertheless, its yield relative to the all-*trans* isomer can be minimised to achieve the objectives of this study.

The key nutrients identified from the screening study were then further optimised using a CCF design and RSM to determine the ideal fermentation media to enhance the production of the biologically significant MK-7 isomer and reduce the yield of the inactive *cis* isomer. The range of all the factors was altered slightly to reposition the design space near the probable optimum. Table 3 illustrates the CCF design matrix and the observed and predicted all-*trans* and *cis* MK-7 isomer concentrations corresponding to each sample. The statistical analysis for the optimisation study is outlined in Table 4. Polynomial regression models were developed based on the significant model terms to predict the yield of the all-*trans* (Eq. 2) and *cis* (Eq. 3) MK-7 isomers as a function of the glucose, yeast extract, soy peptone, tryptone, and CaCl_2_ concentrations.

**Table 3:** Experimental design and responses from the optimisation study

	Nutrient factors (% *w/v*)					Observed responses (mg/L)		Predicted responses (mg/L)	
Run	Glucose	Yeast extract	Soy peptone	Tryptone	CaCl_2_	All-*trans* MK-7 concentration	*Cis* MK-7 concentration	All-*trans* MK-7 concentration	*Cis* MK-7 concentration
1	0.1	0.1	0.1	0.1	1	2.984	0.585	3.643	0.641
2	2	0.1	0.1	0.1	0.1	7.892	1.078	8.403	1.076
3	0.1	2	0.1	0.1	0.1	10.306	1.254	11.113	1.338
4	2	2	0.1	0.1	1	22.264	2.437	22.222	2.444
5	0.1	0.1	2	0.1	0.1	5.605	0.843	5.521	0.829
6	2	0.1	2	0.1	1	20.102	2.545	19.169	2.453
7	0.1	2	2	0.1	1	15.006	1.791	14.368	1.785
8	2	2	2	0.1	0.1	24.138	2.639	23.353	2.575
9	0.1	0.1	0.1	2	0.1	6.345	0.922	7.607	1.003
10	2	0.1	0.1	2	1	17.065	2.091	17.478	2.094
11	0.1	2	0.1	2	1	10.233	1.265	10.942	1.355
12	2	2	0.1	2	0.1	18.555	2.172	19.116	2.203
13	0.1	0.1	2	2	1	10.708	1.407	10.525	1.398
14	2	0.1	2	2	0.1	33.937	3.512	33.606	3.445
15	0.1	2	2	2	0.1	19.399	1.223	19.364	1.242
16	2	2	2	2	1	27.300	1.635	26.416	1.577
17	0.1	1.05	1.05	1.05	0.55	10.343	0.980	7.846	0.679
18	2	1.05	1.05	1.05	0.55	17.190	1.471	18.681	1.713
19	1.05	0.1	1.05	1.05	0.55	14.031	1.109	12.718	1.152
20	1.05	2	1.05	1.05	0.55	17.528	1.452	17.835	1.350
21	1.05	1.05	0.1	1.05	0.55	18.174	1.521	13.296	1.171
22	1.05	1.05	2	1.05	0.55	15.898	1.274	19.771	1.565
23	1.05	1.05	1.05	0.1	0.55	17.384	1.467	17.890	1.498
24	1.05	1.05	1.05	2	0.55	24.060	1.734	22.548	1.645
25	1.05	1.05	1.05	1.05	0.1	26.125	1.738	24.220	1.670
26	1.05	1.05	1.05	1.05	1	22.905	1.666	23.805	1.675
27	1.05	1.05	1.05	1.05	0.55	16.755	1.218	18.375	1.336
28	1.05	1.05	1.05	1.05	0.55	13.270	1.030	18.375	1.336
29	1.05	1.05	1.05	1.05	0.55	21.077	1.524	18.375	1.336

**Table 4:** Statistical analysis of the optimisation study

All-*trans* MK-7 concentration			
Term	Coefficient	Standard error	*P-*value
Constant	18.375	1.156	2.454e-07
*X*_1_	5.417	0.828	1.803e-04
*X*_2_	2.559	0.828	0.015
*X*_3_	3.237	0.828	0.004
*X*_4_	2.329	0.828	0.023
*X*_5_	− 0.207	0.828	0.809
*X*_*1*_^*2*^	− 5.112	2.244	0.052
*X*_2_^2^	-3.099	2.244	0.205
*X*_3_^2^	− 1.842	2.244	0.435
*X*_4_^2^	1.844	2.244	0.435
*X*_5_^2^	5.637	2.244	0.036
*X*_1_*X*_2_	– 1.003	0.879	0.287
*X*_1_*X*_3_	1.178	0.879	0.217
*X*_1_*X*_4_	0.605	0.879	0.511
*X*_1_*X*_5_	0.308	0.879	0.735
*X*_2_*X*_3_	− 0.724	0.879	0.434
*X*_2_*X*_4_	− 1.731	0.879	0.084
*X*_2_*X*_5_	0.333	0.879	0.715
*X*_3_*X*_4_	1.109	0.879	0.242
*X*_3_*X*_5_	− 1.213	0.879	0.205
*X*_4_*X*_5_	− 1.584	0.879	0.109

*Cis* MK-7 concentration			
Term	Coefficient	Standard error	*P*-value
Constant	1.336	0.088	3.501e-07
*X*_1_	0.517	0.063	3.635e-05
*X*_2_	0.099	0.063	0.156
*X*_3_	0.197	0.063	0.014
*X*_4_	0.073	0.063	0.277
*X*_5_	0.002	0.063	0.972
*X*_1_^2^	− 0.140	0.171	0.437
*X*_2_^2^	− 0.085	0.171	0.633
*X*_3_^2^	0.032	0.171	0.854
*X*_4_^2^	0.235	0.171	0.205
*X*_5_^2^	0.337	0.171	0.084
*X*_*1*_*X*_*2*_	− 0.132	0.067	0.083
*X*_*1*_*X*_*3*_	0.082	0.067	0.254
*X*_1_*X*_4_	0.023	0.067	0.740
*X*_1_*X*_5_	− 0.094	0.067	0.199
*X*_*2*_*X*_*3*_	− 0.217	0.067	0.012
*X*_2_*X*_4_	− 0.294	0.067	0.002
*X*_2_*X*_5_	− 0.027	0.067	0.696
*X*_3_*X*_4_	− 0.071	0.067	0.319
*X*_3_*X*_5_	− 0.112	0.067	0.133
*X*_4_*X*_5_	− 0.186	0.067	0.024

*X*_1_ = glucose; *X*_2_ = yeast extract; *X*_3_ = soy peptone; *X*_4_ = tryptone; *X*_5_ = CaCl_2_; *R*^2^ = 0.929; *R*^2^ (adj.) = 0.753; significance accepted at *P* < 0.1

*X*_1_ = glucose; *X*_2_ = yeast extract; *X*_3_ = soy peptone; *X*_4_ = tryptone; *X*_5_ = CaCl_2_; *R*^2^ = 0.948; *R*^2^ (adj.) = 0.817; significance accepted at *P* < 0.1

2 $${Y}_{1}=18.375+5.417{X}_{1}+2.559{X}_{2}+3.237{X}_{3}+2.329{X}_{4}-5.112{X}_{1}^{2}+5.637{X}_{5}^{2}-1.731 {X}_{2}{X}_{4}$$

3 $${Y}_{2}=1.336+0.517{X}_{1}+0.197{X}_{3}+0.337{X}_{5}^{2}-0.132{X}_{1}{X}_{2}-0.217{X}_{2}{X}_{3}-0.294{X}_{2}{X}_{4}-0.186{X}_{4}{X}_{5}$$

Where *Y*_1_ represents the concentration of the all-*trans* MK-7 isomer; *Y*_2_ corresponds to the *cis* isomer concentration; and *X*_1_, *X*_2_, *X*_3_, *X*_4_, and *X*_5_ refer to glucose, yeast extract, soy peptone, tryptone, and CaCl_2_, respectively.

The linear term for glucose (*X*_1_) is especially significant (*P* < 0.001) for both responses, which suggests that the concentration of glucose has a direct relationship with MK-7 production in this particular media. This may be attributed to the critical role of glucose in facilitating microbial growth and metabolism and, consequently, MK-7 production. Additionally, the quadratic terms *X*_1_^2^ and *X*_5_^2^ and the interactive term *X*_2_*X*_4_ were significant (*P* < 0.1) for the all-*trans* MK-7 isomer concentration, whereas the quadratic term *X*_5_^2^ and the interactive terms *X*_1_*X*_2_, *X*_2_*X*_3_, *X*_2_*X*_4_, and *X*_4_*X*_5_ were significant (*P* < 0.1) for the *cis* MK-7 isomer concentration.

The ANOVA analysis (Table 5) demonstrated that the developed models were consistent with the experimental results, as the standard deviation of the regression was considerably greater than the standard deviation of the residuals for both the all-*trans* and *cis* MK-7 isomer concentrations. This is also evident from the significant *P*-value (0.011 and 0.004) and high *F*-value (5.267 and 7.271) for each response. An *R*^2^ value of 0.929 for the all-*trans* MK-7 response and 0.948 for the *cis* MK-7 response indicate a good model fit, as only 7.1% and 5.2% of the total variation, respectively, is not explained by the proposed models.

**Table 5:** ANOVA for the quadratic models

All-*trans* MK-7 concentration						
Source of variation	DF	SS	MS	*F*-value	*P*-value	SD
Total corrected	28	1399.696	49.989	**–**	**–**	7.070
Regression	20	1300.898	65.045	5.267	0.011	8.065
Residual	8	98.798	12.350	**–**	**–**	3.514

*Cis* MK-7 concentration						
Source of variation	DF	SS	MS	*F*-value	*P*-value	SD
Total corrected	28	10.971	0.392	–	–	0.626
Regression	20	10.399	0.520	7.271	0.004	0.721
Residual	8	0.572	0.072	–	–	0.267

*DF* degree of freedom, *SS* sum of squares, *MS* mean sum of squares, *SD* standard deviation

**Fig. 4:**
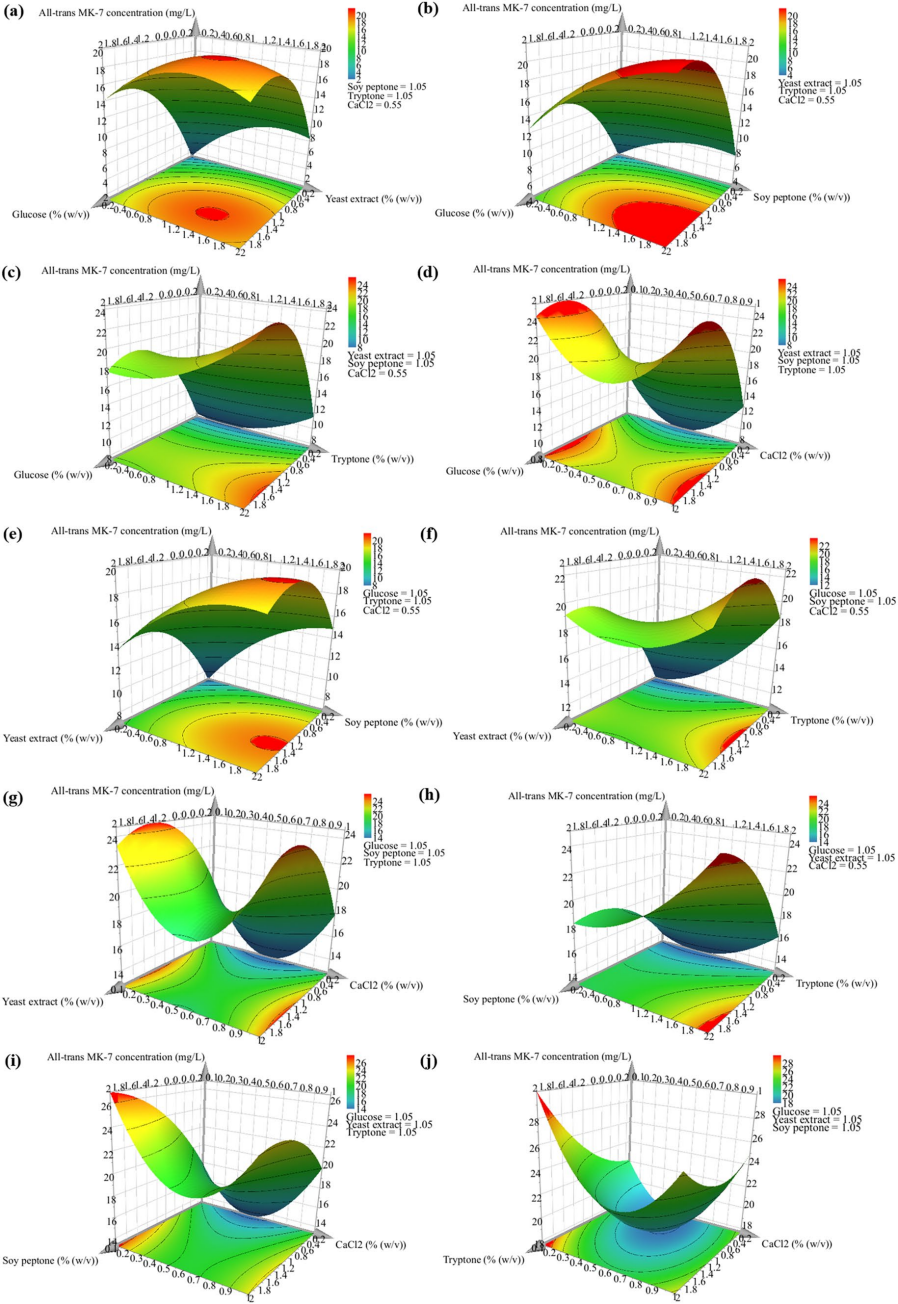
Response surface plots **a-j** demonstrating the effect of each pair of nutrients on the all-*trans* MK-7 isomer concentration

**Fig. 5:**
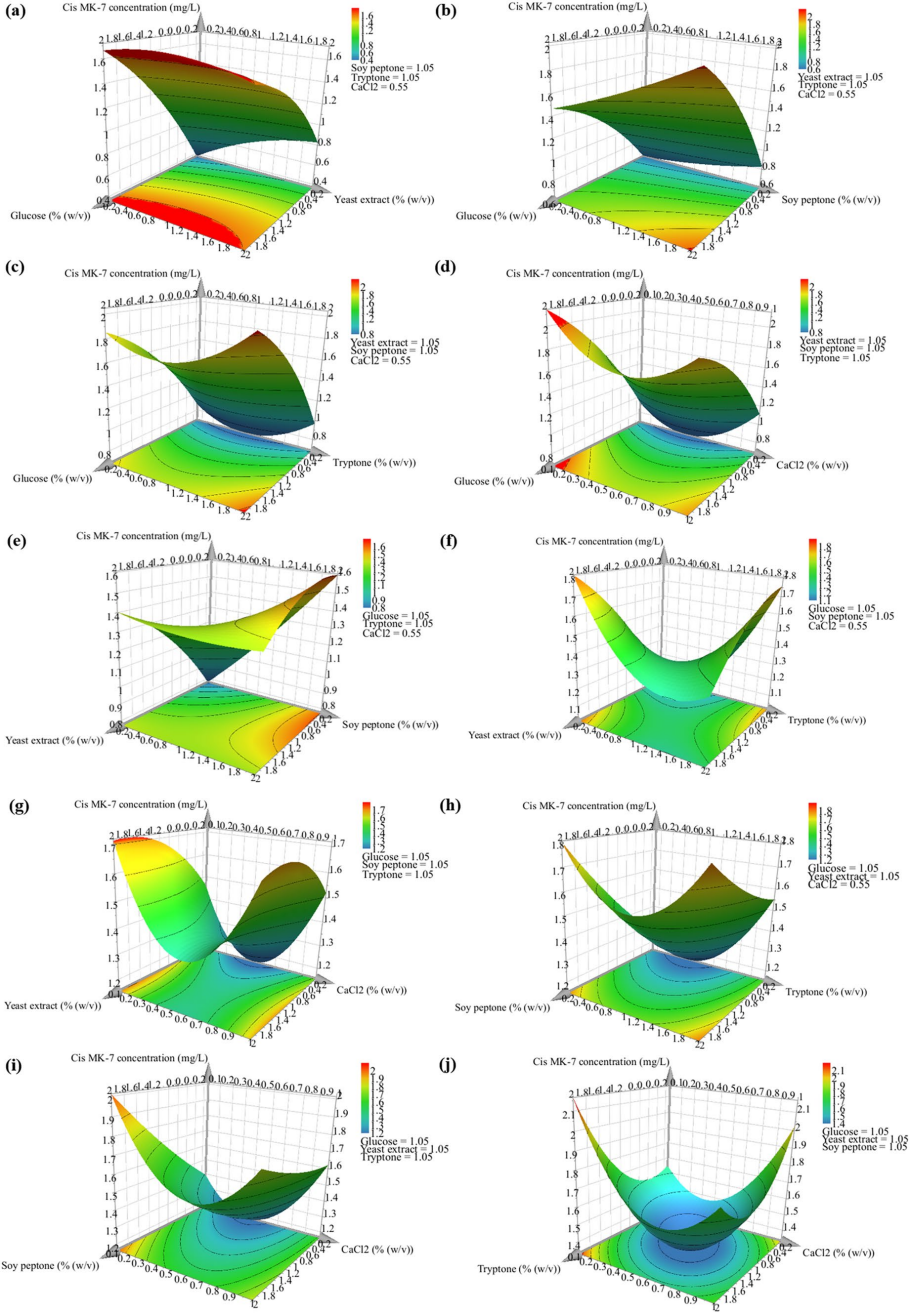
Response surface plots **a-j** demonstrating the effect of each pair of nutrients on the *cis* MK-7 isomer concentration

Interactions between the different media components and their effect on the production of MK-7 isomers can be visualised using contour plots for each pair of nutrients, while the concentration of the remaining nutrients is fixed at their intermediate value. Ten contour plots were generated for each response, and these are illustrated in Fig. 4 for the all-*trans* isomer concentration and Fig. 5 for the *cis* isomer concentration. Overall, the response surface plots depict a complex scenario, as the two responses are interrelated. The objective of this study was to enhance the all-*trans* isomer yield and decrease the production of the *cis* isomer, and the response surface plots demonstrate an intermediate glucose concentration, high yeast extract, soy peptone, and tryptone concentration, and low CaCl_2_ concentration best satisfy this aim.

### Validation study

The ideal concentration of the media components to promote the synthesis of the all-*trans* isomer and reduce the production of *cis* MK-7 was determined by solving the regression equations within the design space. The optimum media contained 1% (*w/v*) glucose, 2% (*w/v*) yeast extract, 2% (*w/v*) soy peptone, 2% (*w/v*) tryptone, and 0.1% (*w/v*) CaCl_2_, and the experimental concentrations (mean ± standard deviation (SD)) of all-*trans* and *cis* MK-7 achieved using the optimised media from triplicate samples were 36.366 ± 2.232 mg/L and 1.225 ± 0.063 mg/L, respectively. Although the experimental concentrations differed from the values envisaged by the model (30.109 mg/L of all-*trans* MK-7 and 1.941 mg/L of *cis* MK-7), the all-*trans* isomer concentration was higher and the *cis* isomer concentration was lower than the concentrations anticipated by the model. Thus, the experimental observations were superior to the model prediction, and the overall results were in accordance with the fundamental aim of this experiment, which was to maximise the production of the all-*trans* isomer and minimise the concentration of *cis* MK-7.

The development of an optimised media to enhance the production of the bioactive MK-7 isomer and decrease the concentration of the inactive isomer is significant from a commercial outlook, as it will improve the process productivity and reduce or eliminate the steps involved in the removal of the *cis* isomer.

### Monitoring the isomer composition and the fermentation process using the optimal fermentation media

**Fig. 6:**
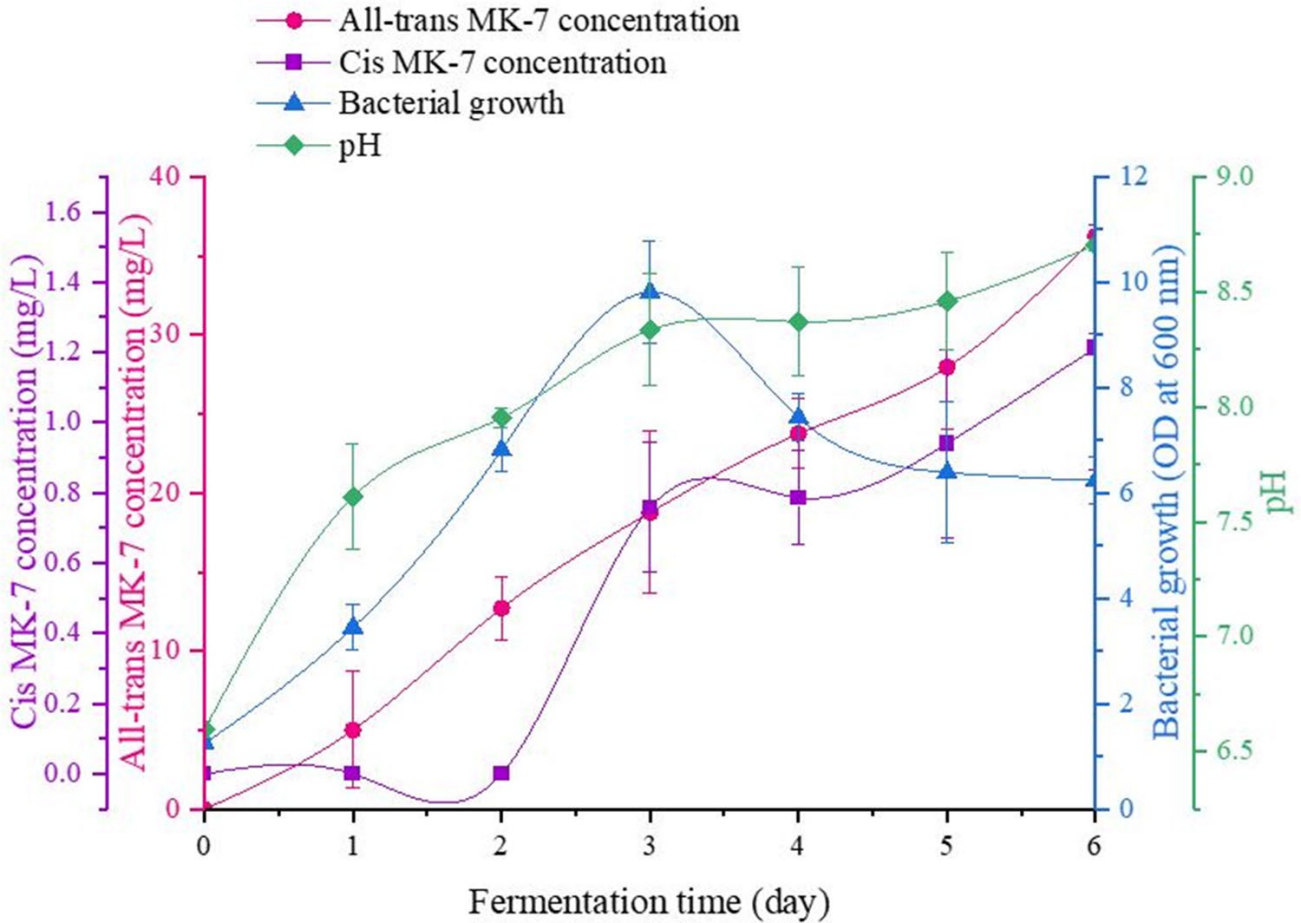
Changes in the all-*trans* and *cis* MK-7 isomer concentrations, bacterial growth, and pH over the fermentation period for the optimised media, which comprised 1% (*w/v*) glucose, 2% (*w/v*) yeast extract, 2% (*w/v*) soy peptone, 2% (*w/v*) tryptone, and 0.1% (*w/v*) CaCl_2_ (the error bars represent the SD calculated from four replicate samples for each response)

The all-*trans* and *cis* MK-7 isomer concentrations, bacterial growth, and pH were analysed each day over the course of fermentation for the optimised media (Fig. 6). The trends observed in MK-7 isomer production, particularly the all-*trans* isomer, closely reflected the cell growth pattern, congruous with accounts from previous investigations [61, 84, 89, 97]. The OD increased slowly over the first day of fermentation, after which it escalated to 9.81 on day 3 and decreased to 7.43 on day 4, ultimately reaching a value of 6.24 on day 6. The variation in the OD measurements corresponds to the bacterial growth profile, and days 0 to 1 are likely to represent the lag phase, between days 1 and 3 correlate with the exponential growth period, and days 4 to 6 appear to denote the stationary phase.

It is interesting to note that although the overall variation in the concentration of the two isomers over the investigated timeframe is similar, the day on which their production began was different. Production of the all-*trans* isomer commenced on day 1 and increased over the fermentation period at a rate compliant with the bacterial growth profile, reaching a final concentration of 36.232 mg/L on day 6. Correspondingly, 13.83% of the total all-*trans* MK-7 was noted during the lag phase, 37.95% was synthesised during the logarithmic growth phase, and 48.22% was produced during the stationary phase. In comparison, production of the *cis* isomer initiated on day 3 and increased according to the bacterial growth curve, resulting in a concentration of 1.214 mg/L at the end of fermentation. Accordingly, 0% of the total *cis* MK-7 was observed during the lag phase, 62.47% was produced during the exponential growth phase, and 37.53% was detected during the stationary period. The delay in the production of the *cis* isomer, relative to all-*trans* MK-7, is an intriguing observation, as it would generally be expected for both isomers to be synthesised simultaneously. There are two probable explanations for this finding. Since the all-*trans* isomer is the naturally occurring form of the vitamin, it is produced in a greater quantity than the *cis* isomer, and thus, the concentration of *cis* MK-7 may be too low to be detected by the instrument. Alternatively, it is possible for the *cis* isomer to be produced from the isomerisation of all-*trans* MK-7 due to the culture or environmental conditions, and this may occur after a few days of fermentation once a sufficient amount of the all-*trans* isomer has been synthesised. The trend in both the all-*trans* and *cis* isomer concentrations seems to continue to increase at the end of the fermentation period, which contrasts with the trend in the OD measurements.

Prior studies [61, 84, 97] have reported that the MK-7 concentration plateaus during the stationary phase, suggesting that MK-7 production correlates with bacterial growth. Whereas Xu and Zhang [92], Sato et al. [87], and Song et al. [63] have observed a substantial rise in the MK-7 concentration when the bacterial culture enters the stationary phase, which may be ascribed to the release of intracellular MK-7 as a result of cell lysis [92]. MK-7 is synthesised intracellularly and is secreted from the cell as a soluble complex with an acidic binding factor [92, 95, 98–100]. It has been noted that the amount of MK-7 inside *B. subtilis* cells is greater than the extracellular quantity [53, 92]. Therefore, during the stationary phase, it is anticipated for the extracellular MK-7 concentration to rapidly increase due to cell rupture and the successive release of the intracellular contents [92]. However, when bacterial growth enters the death phase, the MK-7 concentration is likely to decrease, as over an extended timeframe, proteases, other enzymes, and various cellular components released during cell lysis may degrade the MK-7 present in the fermentation broth. Consequently, it is potentially advantageous to terminate fermentation before the onset of the death phase to ensure maximal all-*trans* MK-7 production.

Consolidating the findings from prior studies and the observations from this investigation, MK-7 is a mixed metabolite, as its production is partly growth-associated. The majority of MK-7 biosynthesis possibly occurs during the logarithmic phase, resulting in a gradual rise in the MK-7 concentration during this stage as some intracellular MK-7 is secreted into the extracellular fraction. A significant increase in the MK-7 concentration may only be noted during the stationary phase when the intracellular MK-7 is released due to cell lysis. Further extension of the fermentation period beyond the stationary phase and into the death phase might not be ideal, as it may lead to a drop in the MK-7 concentration. However, in the present study, it is necessary to prolong fermentation and observe the trends in bacterial growth and MK-7 production beyond day 6 to draw accurate conclusions in this regard.

Additionally, the pH of the media progressively increased from an initial value of 6.60 to 8.71 at the end of the fermentation period. This change in pH is verified by previous investigations [61, 92, 97] and may be attributed to protein hydrolysis and the subsequent release of ammonia by *B. subtilis* [61, 97].

## Conclusions

This study was the first to explore the production of MK-7 isomers in a fermentation context and consider the development of an optimised media to enhance the concentration of the biologically significant all-*trans* isomer and minimise the production of the inactive *cis* isomer. A PBD was initially employed to screen ten different carbon, nitrogen, and salt sources to determine the most effective nutrients that have a notable effect on the MK-7 isomer concentration. Glucose, yeast extract, soy peptone, tryptone, and CaCl_2_ were then further analysed in an optimisation study using a CCF design and RSM to ascertain the optimum fermentation media. An experimental all-*trans* isomer concentration of 36.366 mg/L and a *cis* isomer concentration of 1.225 mg/L were achieved using the ideal fermentation media, which consisted of 1% (*w/v*) glucose, 2% (*w/v*) yeast extract, 2% (*w/v*) soy peptone, 2% (*w/v*) tryptone, and 0.1% (*w/v*) CaCl_2_. The experimental concentrations were superior to the values predicted by the model. This media presents a commercially attractive alternative to MK-7 synthesis using fermentation media that does not specifically target the production of the bioactive isomer and, thus, necessitates the separation and removal of *cis* MK-7 from the desired all-*trans* product. Although the *cis* isomer is inactive, it may not be possible to eliminate its production during fermentation, as it is synthesised in small quantities alongside the all-*trans* isomer. Therefore, MK-7 synthesis using an optimised fermentation media, which enhances the yield of the biologically important all-*trans* isomer and minimises the concentration of *cis* MK-7, is likely to entail fewer downstream processing steps and reduce the costs associated with the industrial fermentation of the vitamin.
